# Abrupt Termination of Severe Treatment Refractory Neuropsychiatric Symptoms in a Patient Treated With Rituximab—A Case Report

**DOI:** 10.1155/crps/6717967

**Published:** 2026-09-18

**Authors:** Daniel Albert, Brian Rosen, Juliette Madan

**Affiliations:** ^1^ Medicine and Pediatrics, Geisel School of Medicine at Dartmouth, Hanover, New Hampshire, USA, dartmouth.edu; ^2^ Psychiatry and Dermatology, Geisel School of Medicine at Dartmouth, Hanover, New Hampshire, USA, dartmouth.edu; ^3^ Psychiatry, Pediatrics and Epidemiology, Geisel School of Medicine at Dartmouth, Hanover, New Hampshire, USA, dartmouth.edu

**Keywords:** autoimmunity, psychosis, rituximab, treatment

## Abstract

A 65‐year‐old woman presented to a joint rheumatology, neurology, and psychiatry clinic with subacute onset of severe neuropsychiatric symptoms that began 2 years prior. The symptomatology had been dominated by paranoid ideation, withdrawal from family, and cognitive changes. Over the previous 2‐year period the patient required inpatient psychiatric care three times with provisional diagnoses, including major depressive disorder with psychotic features, schizophrenia, and late‐onset schizophrenia. Pharmacologic treatments consisted of multiple trials of different antidepressants and antipsychotics, eventually with a trial of clozapine without notable benefit and further worsening of symptoms. Ultimately the patient was unable to perform her activities of daily living (ADL) independently. Past medical history was notable for pernicious anemia. An extensive evaluation was notable for antibodies to intrinsic factor consistent with pernicious anemia and low‐titer antiphospholipid antibodies. Magnetic resonance imaging (MRI), electroencephalography (EEG), and cerebrospinal fluid (CSF) evaluations were all unrevealing. Neuropsychologic testing demonstrated difficulties with executive function and semantic retrieval along with ADL deficits meeting criteria for major neurocognitive disorder (MNCD) of uncertain etiology. A multidisciplinary evaluation ultimately identified a possible systemic lupus erythematosus (SLE)‐related process. The patient received two 1‐g infusions of rituximab. Approximately 3 weeks later she experienced a substantial reduction in her complex neuropsychiatric symptoms, and within 6 months she almost completely returned to her premorbid level of functioning. This case illustrates an atypical presentation of treatment‐resistant neuropsychiatric and neurocognitive symptoms that benefitted from comprehensive, multidisciplinary evaluation and treatment. When frank antibody‐positive autoimmune encephalitis and antibody‐negative autoimmune encephalitis have been excluded, clinicians should consider underlying emerging systemic autoimmune disease, for example, SLE as a potential cause of psychosis and other severe neuropsychiatric manifestations, prompting further diagnostic workup and treatment strategies, including immunotherapy.

## 1. Introduction

While there are a few exceptions, the majority of neuropsychiatric disorders are of unknown etiology [1]. However, some autoimmune diseases, in particular systemic lupus erythematosus (SLE), can have psychiatric manifestations prior to the onset of objective physical features [2]. Furthermore, it was relatively recently discovered that the autoantibody anti‐*N*‐methyl‐D‐aspartate (NMDA) receptor antibody can manifest solely as a psychiatric disorder [3]. Lastly, one of the strongest genetic links to psychiatric disorders is a gene in the complement cascade, which is a strong risk factor for SLE [4]. These immune‐related mechanisms are generally believed to constitute exceptional cases, while the majority of psychiatric cases are still viewed as “etiologically enigmatic.” The following case report illustrates a patient with psychosis, neurocognitive decline, and limited evidence for autoimmunity who dramatically responded to immunotherapy with rituximab.

## 2. Case Presentation

The patient was a 65‐year‐old woman with a subacute onset of psychosis who, after extensive evaluation, had mild and subtle features of autoimmunity. She failed numerous antipsychotic and antidepressant medications and was on the verge of residential care when we empirically tested immunosuppressive therapy with rituximab, with surprising and dramatic improvement.

The patient presented to our joint rheumatology, neurology, and psychiatry clinic for evaluation of treatment‐resistant late‐onset psychotic spectrum illness referred from our mood disorders clinic due to the atypical features (Table 1). Psychiatric symptoms began in her late 50’s with intermittent mild paranoia, which led to her quitting her job. The symptoms appeared to resolve fully before developing an acute worsening of persecutorial delusions with bizarre content consisting of the devil and demons 2 weeks after severe acute respiratory syndrome coronavirus 2 (SARS Co‐V2) infection at age 63 years. The patient was psychiatrically hospitalized following this acute worsening, and on evaluation, symptoms were dominated by paranoid ideation. She was given a preliminary diagnosis of major depressive disorder with psychotic features; however, the patient’s profound abulia, anhedonia, and poverty of thought were considered atypical. The patient followed closely with adult psychiatry and was treated unsuccessfully with citalopram, escitalopram, fluoxetine, sertraline, mirtazapine, topiramate, trazodone, risperidone, quetiapine, zolpidem, olanzapine, and clozapine. She developed choreoathetoid movements consistent with extrapyramidal effects from some of these medications. Electroconvulsive therapy was considered but declined by the patient and her husband. As her course progressed, the “depressive” thought content dissipated, she no longer reported any hallucinations or delusions, and the patient was left with what most closely matched the negative symptoms of schizophrenia. She was given a provisional diagnosis of schizophrenia, although she no longer had the clear positive symptoms of paranoia noted previously. The patient was subsequently seen by an outside institution for a second opinion and was diagnosed with late‐onset schizophrenia. Referral to our joint multidisciplinary clinic occurred when, despite her numerous medication trials, her function and symptoms continued to worsen to the point of being nearly nonverbal, withdrawing completely from interactions with family or any activities of daily living (ADL), in clear distress with screaming, loss of bowel and bladder function, and inability to manage her basic ADLs.

**Table 1:** Timeline.

July 2022	Long standing anxiety, depression, and intermittent mild paranoid ideation but functional at work and ADLs
November 2022	SARS Co‐V2 infection
November 2022	Several weeks after the SARS Co‐V2 infection abrupt decline in cognitive function with worsening paranoia, decreased cognition, staring spells, and poor ADLs resulting in a psychiatric hospital admission
November 2024	Progressive decline despite numerous medication trials with the development of extrapyramidal motor activity labeled tardive dyskinesia. Needed 24‐h care because she could not care for herself.
December 2024	Received first dose of 1‐g rituximab with 100 mg solumedrol. Tolerated well. No discernable improvement.
January 2025	Received second dose of 1‐g rituximab without solumedrol which she tolerated well.
April 2025	Reported dramatic improvement in mental state. No paranoid ideation, cognition mostly back to baseline, speaking clearly, no staring spells, and completely independent for ADLs.

The patient’s past medical history was notable for suspected childhood‐onset seizures that were resolved without medical intervention, latent tuberculosis, and pernicious anemia. She demonstrated a vitamin B12 deficiency secondary to antibodies to intrinsic factor that was corrected with parenteral and oral vitamin B12 supplements and iron deficiency secondary to Heliobacter pylori infection, which had resolved. The family history was notable for a sister with Parkinson’s disease. The review of symptoms for autoimmune disease was negative, and her physical exam was also noncontributory. Laboratory examination was extensive and included serologic evaluation, magnetic resonance imaging (MRI), electroencephalography (EEG), and lumbar puncture. Serologic evaluation was notable for antibodies to intrinsic factor, positive antinuclear antibody at 1:320 dilution, normal serum complement, negative double‐stranded deoxyribonucleic acid (DNA) antibody, and low‐titer antiphospholipid antibodies with no specific clinical features of antiphospholipid syndrome (no miscarriages or thrombosis). The myositis antibody panel was negative. Lyme antibodies and QuantiFERON gold tuberculosis tests were negative. MRI showed mild scattered white‐matter lesions consistent with age or hypertension and a congenital venous anomaly. EEG wave patterns were nonspecific. Cerebrospinal fluid (CSF) evaluation showed no increased protein or cells. CSF autoimmune and paraneoplastic panels were negative. Serial Montreal cognitive assessment (MOCA) screening tests were obtained throughout the clinical course, consistently scoring 29/30 [5]. Neuropsychological testing demonstrated difficulties with learning and memory, semantic retrieval, and executive functioning. Given the profound deficits, the patient was given a diagnosis of major neurocognitive disorder (MNCD) of unclear etiology, although the report could not determine what role the psychotic spectrum symptoms were playing and noted the atypical pattern of dysfunction.

Given the atypical course of the patient’s symptoms and subacute worsening of instrumental ADLs (iADLs) and ADLs, a diagnosis of seronegative autoimmune encephalitis was considered. She received infusions of 1000 mg of rituximab starting in December 2024 and again 3 weeks later. The first infusion included 100 mg of solumedrol. Approximately 3 weeks after the second infusion, the patient had a dramatic improvement with amelioration of negative symptoms with some functional improvement. She redeveloped social graces/reciprocity, began to watch television, and read books voraciously, achieving almost complete return to her premorbid function within 6 months. Repeated neuropsychologic testing demonstrated an “intact cognitive profile,” stating that she no longer met criteria for MNCD. As of May 2026, she is driving independently and plans to renew her volunteer activities. Her husband reports that it is hard to express how miraculous the changes have been.

## 3. Discussion

Patients with acute or subacute onset of neuropsychiatric symptoms that are atypical or treatment‐refractory warrant multidisciplinary evaluation and treatment. Despite intense investigation and some improvement in therapies, the etiologies of severe, chronic psychiatric conditions often remain unclear. Genome‐wide association studies have identified hundreds of susceptibility loci (including a key gene of autoimmunity in the complement 4 locus), suggesting genetic components for psychotic illnesses [4, 6]. However, there is little suggestion of a specific genetic causal pathway, and statistical analysis of genetic studies suggests a large component of environmental influences [6]. In several autoimmune disorders, including SLE, Behcet’s syndrome, and multiple sclerosis, neuropsychiatric manifestations often precede the formal diagnosis [2]. Epidemiologic features of primary or idiopathic psychiatric disorders, including gender, age, and exposure to possible triggering events closely mirror those of autoimmune diseases [7, 8]. Recent data on streptococcus and other infection‐triggered neuropsychiatric diseases in children, that is, pediatric acute‐onset neuropsychiatric syndrome (PANS) and pediatric autoimmune neuropsychiatric disorders associated with streptococcal infections (PANDAS), suggest a potential mechanism for the induction of autoimmunity that present as psychiatric diseases [7–9]. Case reports of prolonged psychiatric disorders such as catatonia or severe obsessive compulsive disorders (OCDs) [10] reversing with immunosuppressive medications, as well as the dramatic reversal observed in NMDA receptor antibody disease (“brain on fire”), highlight the connection [11].

The consensus guidelines for autoimmune psychosis, published in the Lancet psychiatry in 2020, outline objective criteria and highlight the possibility that specific presenting symptoms and markers of inflammation and autoimmunity, even in the absence of frank autoimmune disease, warrant evaluation and multidisciplinary treatments [12]. Whether our patient fits into the diagnostic criteria for autoimmune psychosis or even less likely autoimmune encephalitis is somewhat equivocal and depends on whether certain features correspond to the criteria (Table 2). For example, did she have a movement disorder? Our interpretation was that she had tardive dyskinesia from the medication rather than from her underlying basal ganglia disorder. She had adverse reactions to some antipsychotics and little or no improvement. Consequently, she did not meet the current diagnostic criteria for either seronegative autoimmune encephalitis or autoimmune psychosis [13, 14]. The current consensus approaches to the diagnosis of autoimmune encephalitis or autoimmune psychosis likely do not capture all the patients who present with severe neurobehavioral abnormalities who may benefit from immunomodulatory medications. This patient is an example of a patient who clearly had a serious neuropsychiatric and neurocognitive disorder with a robust response to immunosuppressive medication but whose findings fell outside of existing diagnostic frameworks. Neuroimmune processes in a younger cohort of patients have been reported, where atypical treatment‐refractory neuropsychiatric symptoms dominated, autoimmune features were frequent, and adjunctive immunotherapy provided benefit [15].

**Table 2:** Features and diagnoses of autoimmune neurobehavioral disorders.

Features of autoimmune encephalitis
1. Subacute onset (<3 months)
2. At least one of the following
a) New focal CNS findings
b) New‐onset seizures
c) CSF pleocytosis
d) MRI findings of encephalitis
3. Exclude other possibilities
Features of possible autoimmune psychosis
1. Subacute onset (<3 months)
2. At least one of the following
a) Recent diagnosis of a tumor (benign or malignant)
b) Movement disorder
c) Adverse response to antipsychotics
d) Decreased level of consciousness
e) New‐onset seizures
f) New‐onset autonomic neuropathy
Diagnostic criteria for probable autoimmune psychosis
1. Abrupt onset psychosis (<3 months)
2. One of the seven clinical criteria listed above and at least one of the following:
a) CSF pleocytosis
b) Brain abnormalities on MRI
c) EEG changes consistent with encephalopathy
d) CSF oligoclonal bands or increased IgG index
e) Antineuronal antibody
Definite autoimmune psychosis
1. Probable autoimmune psychosis
2. IgG‐class antineuronal antibodies
Our patient
1. Possible abrupt onset
2. No tumor was identified
3. No movement disorder (unless you count tardive dyskinesia)
4. Poor response to antipsychotics but not clearly adverse response
5. No depressed level of consciousness
6. No seizure
7. No autonomic neuropathy
8. No CSF pleocytosis
9. No brain MRI findings of encephalitis
10. No EEG changes in c (w) encephalitis
11. No CSF oligoclonal bands or increased IgG index
12. No antineuronal antibody

The scope of patients with disabling neuropsychiatric conditions secondary to an immune etiology is unknown. Schizophrenia alone affects 1% of the population [16]. Even if a small proportion of these patients have an autoimmune pathogenesis and may benefit from immunosuppressive medication [17], the public health implications would be substantial. Early identification is a priority. Red‐flag symptoms include abrupt onset, especially with cognitive decline and motor features, personal or family history of autoimmunity, and failure to respond to conventional therapies [12, 18]. Patients for whom atypical age of onset, rapid symptom presentation, additional atypical features, and lack of response to treatment warrant thorough multidisciplinary evaluation for autoimmunity that includes serology (especially autoimmune encephalitis panel antibodies and antiphospholipid antibodies) [19], MRI, EEG, and CSF analysis, and, in select patients, genetic testing for immune disorders. A recent open‐label study of rituximab for patients with OCD and schizophrenia spectrum disorder demonstrated measurable improvements [20], prompting a planned protocol for a randomized controlled trial of rituximab for psychotic disorders [21].

## 4. Conclusions

The case presented here illustrates an atypical presentation of treatment‐resistant neuropsychiatric and neurocognitive symptoms that benefitted from comprehensive, multidisciplinary evaluation and treatment. When frank antibody‐positive and ‐negative autoimmune encephalitis have been excluded, clinicians should consider underlying emerging systemic autoimmune disease, for example, SLE and other autoimmune and autoinflammatory conditions, as a potential cause of psychosis and other severe neuropsychiatric manifestations, prompting further diagnostic workup and treatment strategies including immunotherapy.

## Funding

No funding was received for this manuscript.

## Consent

The patient gave written consent to write, submit, and publish this report.

## Conflicts of Interest

The authors declare no conflicts of interest.

## Data Availability

The data that support the findings of this study are available upon request from the corresponding author. The data are not publicly available due to privacy or ethical restrictions.
